# The Impact of Arp2/3 Complex Inhibition on Cytoskeleton Dynamics and Mitochondrial Function during Goat Oocyte Meiosis

**DOI:** 10.3390/ani13020263

**Published:** 2023-01-12

**Authors:** Meng-Hao Pan, Rui Xu, Yiqian Zhang, Lu Yin, Ruoyu Li, Dongxu Wen, Sihai Lu, Yan Gao, Xiaoe Zhao, Qiang Wei, Bin Han, Baohua Ma

**Affiliations:** 1 College of Veterinary Medicine, Northwest A&F University/Key Laboratory of Animal Biotechnology, Ministry of Agriculture, Yangling 712100, China; 2 Yulin Animal Husbandry and Veterinary Service Center, Yulin 719000, China

**Keywords:** ARP2/3, goat oocyte, actin, spindle, mitochondria

## Abstract

**Simple Summary:**

The actin-related protein complex 2/3 complex (Arp2/3) is a kind of actin regulatory protein that regulates the nucleation, aggregation and assembly of F-actin. In recent years, there have been reports at home and abroad about the effects of the Arp2/3 complex on oocyte maturation of mammals such as pigs and mice, but few studies are about its effects on goats. In present study we showed that the Arp2/3 complex is necessary for the maintenance of cytoskeletal meiotic dynamics, such as F-actin assembly and spindle organization in goat oocytes. Additionally, these cytoskeletal dynamics are essential for the regulation of mitochondrial distribution and function, including mitochondrial activity and redox homeostasis, which are critical for the meiotic maturation of goat oocytes.

**Abstract:**

F-actin is of critical importance in oocyte meiotic maturation. Actin assembly and its dynamics are mainly regulated by actin nucleation factors. The actin-related protein complex 2/3 (Arp2/3) is responsible for the organization of F-actin filaments. However, the role of Arp2/3 complex in goat oocytes has not been fully elucidated. Our findings demonstrate that Arp2/3 complex activity is necessary for the maturation of goat oocytes. The Arp2/3 complex-specific inhibitor CK666 impairs the maturation of goat oocytes and alters the genes associated with cumulus expansion, both of which suggest that normal meiosis is affected. Arp2, one of the subunits of the Arp2/3 complex, was found to be mainly accumulated at the oocyte cortex and to co-localize with F-actin during goat oocyte maturation in our results. Thus, we further investigated the cytoskeleton dynamics and found that Arp2/3 complex inhibition disrupts the F-actin assembly and spindle organization. Further analysis revealed that, in addition to direct effects on the cytoskeleton, Arp2/3 complex could also induce ROS accumulation and oxidative stress by disrupting mitochondrial distribution and function, ultimately increasing the rate of early apoptosis in goat oocytes. Our study provides evidence that the Arp2/3 complex is a key regulator of goat oocyte maturation through its regulation of the cytoskeleton dynamics and mitochondrial function.

## 1. Introduction

In the breeding industry, the reproductive capacity of livestock has a close bearing on the economic benefits of animal production, and the quality of female oocytes is the key to its evaluation. F-actin plays a significant role in the meiosis of female mammalian oocytes [1]. On the one hand, F-actin regulates the long-distance transport of vesicles in oocytes [2]. On the other hand, F-actin also play other important roles, including the regulation of spindle migration and positioning during oocyte meiosis, the regulation of the cortical polarization of oocytes, the regulation of polar body excretion, the regulation of chromosome segregation and capture during oocyte meiosis, and the regulation of organelle distribution and the interaction of gamete-somatic cells during oocyte meiosis to regulate oocyte maturation, according to the latest research report [1,3]. 

The importance of F-actin in oocyte meiotic maturation cannot be understated, with assembly and dynamics primarily regulated by actin nucleators [4,5]. The actin-related proteins 2/3 (Arp2/3) complex is a key player in eukaryotic cell biology, capable of organizing actin filaments into intricate, branched networks. This complex is composed of two primary subunits, Arp2 and Arp3, as well as five additional Arpc subunits (Arpc1-Arpc5). Together, these seven subunits form a powerful actin-nucleating machine that is essential for many cellular processes [6,7,8]. The conformation of the Arp2/3 complex in which Arp2 and Arp3 subunits are arranged far apart from each other is known as the inactive state, the active state Arp2/3 complex on the other hand, features the rearrangement of these subunits into proximity, which mimics the end of an actin filament [9]. By interacting with a preexisting actin filament, the active conformation of Arp2/3 complex can initiate the elongation of a lateral branch [10]. The Arp2/3 complex also interacts with nucleation-promoting factors (NPFs) to mediate the formation of branched-chain actin networks; this is necessary for processes such as cytoskeletal remodeling, intracellular trafficking and cell motility [11]. 

Numerous studies have demonstrated the significance of the Arp2/3 complex in cell function. Inhibition of the Arp2/3 complex’s activity has far-reaching consequences for the cell, including the disruption of migration, adhesion, endocytosis, and the establishment of cell polarity during mitosis [12]. In epithelia tissues, the activity of the Arp2/3 complex is necessary for epithelial cell function and tissue morphogenesis [13]. The Arp2/3 complex has been found to be a key player in mouse oocyte asymmetric division [14,15]. According to studies, the Arp2/3 complex plays a vital role in the meiotic process of mouse oocytes by regulating the movement of the spindle and the formation of the actin cap. This complex generates the hydrodynamic force required for the spindle to move toward the oocyte cortex, as well as regulating cytokinesis to ensure proper cell division [14,16]. The arrested spindle migration is the primary factor responsible for the equal division in mouse oocytes. In comparison to mouse oocytes, the germinal vesicle of cow, pig, and horse oocytes is generally eccentric, as a consequence, the radial migration of the MI spindle is shortened [17]. The research on porcine oocytes revealed that Arp2/3 had no influence on the asymmetric division of oocytes, but also plays a crucial role in porcine cumulus proliferation and oocyte maturation [18]. 

The effects of the Arp2/3 complex on oocyte maturation have been widely studied in mammals such as pigs and mice, however, there is little research on its effects on goats. Various questions are still worth investigating such as whether the Arp2/3 complex influences goat oocyte maturation, the specific mechanism by which the Arp2/3 complex regulates goat oocyte maturation, and whether its expression in goats is different from other species. 

## 2. Materials and Methods

### 2.1. Antibodies

Phalloidin-FITC, and mouse monoclonal anti-α-tubulin-FITC antibodies were obtained from Sigma-Aldrich Corp (St. Louis, MO, USA; Cat# SML0006, P5282, F2169); Phalloidin-TRITC was obtained from Invitrogen (Carlsbad, CA, USA; Cat# R415); Rabbit polyclonal anti-Arp2 antibody was obtained from Bioss (Beijing, China; Cat# bs-12524R); all other chemicals utilized in this study were sourced from Sigma (St. Louis, MO, USA), unless otherwise stated.

### 2.2. COCs Collection and In Vitro Maturation

All animal experimentation was performed under the auspices of the institutional Animal Care and Use Committee of the college of Veterinary Medicine at Northwest A&F University. Ovaries were collected from goats at a local abattoir (Yulin, China). The goat ovary should be placed into a sterile solution of normal saline that contains both penicillin and streptomycin. Afterwards, they can be placed into thermos bottles and transported to the laboratory within a 1–4 h time period. Once the goat ovary was brought back to the laboratory, the nearby tissues were removed and the ovary was washed with normal saline. The observable antral follicles on the surface of the ovary were punctured with the tip of a scalpel blade. The ovary was then gently squeezed to ensure that the COCs followed the follicular fluid. 

Oocytes with an intact and well-formed compact cumulus mass were selected for in vitro maturation. The in vitro maturation (IVM) medium was composed of the TCM-199 (Gibco, Carlsbad, CA, USA; Cat# 11043023) supplement with 1 mM Na-pyruvate, 2.5 mM glutamine, 1% (*v/v*) NEAA, 1% (*v/v*) ITS, 10% (*v***/***v*) FBS, 1 μg/mL Estradiol, 0.075 IU/mL HMG, 10 ng/mL EGF, 75 μg/mL penicillin and 50 μg/mL streptomycin. The selected germinal vesicle (GV) COCs were transferred into a four-well dish containing 500 uL of IVM medium. The COCs were incubated at 38.5 °C with 5% CO2 for 12 h or 24 h, depending on whether the goal was to reach the metaphase I stage (MI) or the MII stage. 

### 2.3. CK666 Treatment

CK666 (Sigma-Aldrich Corp, St. Louis, MO, USA; Cat# SML0006) was dissolved in DMSO to create a 100 mM reserve solution. For the pre-experiment, we used a working concentration of 50 μM, 100 μM and 20 μM in IVM medium. Comparative to the pre-experiment results, we chose 100 μM for following experiment. Thus, in CK666 group, COCs were cultured in 100 μM CK666 concentration, and in control group, COCs were cultured in 1‰ DMSO. Culturing COCs in IVM medium with CK666 inhibitor for 12 h allowed for the evaluation of F-actin assembly, spindle composition, mitochondrial activity and oocyte apoptosis in goat oocytes, whereas 24 h of culturing was used to determine the oocyte maturation rate of goat oocytes. After IVM, to obtain the denuded oocytes, the removal of cumulus cells surrounding the COCs was carried out gently with a fine-bore pipette in 0.5% hyaluronidase solution (in IVM medium).

### 2.4. Immunofluorescence Staining 

Oocytes were fixed in 4% paraformaldehyde (in phosphate-buffered saline (PBS)) for 30 min, then permeabilized with 1% Triton X-100 in PBS for 1 hour, and finally blocked in blocking buffer (1% BSA-supplemented PBS) at room temperature for 1 h and set aside. Arp2 staining was completed by incubating oocytes that were blocked with primary antibody (1:200) at 4 °C overnight, then washed 3 times by PBST (0.1% Tween 20 and 0.01% Triton X-100 in PBS) and incubated with Alexa Fluor 488 goat anti-rabbit (1:200) for 1 h at room temperature. For cytoskeleton staining, the oocytes were incubated with an anti-α-tubulin-FITC antibody (1:100) at 4 °C for a period of one night in order to facilitate the α-tubulin staining; oocytes were incubated with either Phalloidin-TRITC or Phalloidin-FITC at room temperature for 2 h following the completion of blocking, in order to observe actin staining. Oocytes were incubated with Hoechst 33342 at room temperature for 15 min in order to evaluate nuclear morphology. After staining, the samples were observed with a confocal laser-scanning microscope (Nikon Eclipse Ti, Japan; Leica TCS SP8, Germany). 

### 2.5. Mito-Tracker Staining

Mito-Tracker Red CMXRos (Beyotime, Shanghai, China; Cat# C1035) was used to investigate the localization of mitochondria in oocytes. The MI stage oocytes were transferred from IVM medium to Mito-Tracker red ((1:800, with IVM medium dilution)) for 30 min at 38.5 °C and 5% CO_2_. Following three washes with mDPBS, the oocytes were examined using a Fluorescent microscope (OLYMPUS IX71, Tokyo, Japan) for the presence of a fluorescent signal.

### 2.6. JC-1 Detection

An enhanced mitochondrial membrane potential assay Kit (Beyotime, Shanghai; Cat# C2003S); was employed to analyze the mitochondrial membrane potential of the oocytes. The MI stage oocytes were transferred from the IVM medium to JC-1for 30 min at 38.5 ℃. Following three washes with mDPBS, the oocytes were examined using a Fluorescent microscope (OLYMPUS IX71, Japan) for the presence of a fluorescent signal.

### 2.7. Reactive Oxygen Species (ROS) Detection

The Reactive Oxygen Species Assay Kit (Beyotime, Shanghai, China; Cat# S0033S); was used to measure the ROS levels in oocytes. The MI stage oocytes were transferred from the IVM medium to a solution of DCFH-DA (1:800 dilution with IVM medium) for 30 min at 38.5 °C. Following three washes with mDPBS, the oocytes were examined using a Fluorescent microscope (OLYMPUS IX71, Japan) for the presence of the ROS fluorescent signal.

### 2.8. Annexin-V Staining

The oocytes were washed twice in PBS and then stained with Annexin-V-FITC (Solarbio, Beijing, China; Cat# CA1020) in binding buffer for 30 min at 38.5 ℃ in the dark. Following three washes with mDPBS, the oocytes were examined using a Fluorescent microscope (OLYMPUS IX71, Japan) for the presence of the fluorescent signal using the same scanning settings.

### 2.9. Quantitative Real-Time PCR Analysis 

The MiniBEST Universal RNA Extraction Kit (Takara, Japan; Cat# 9767) was utilized to lyse COCs and extract total RNA. PrimeScript RT Master Mix (Takara, Japan; Cat# RP036Q) was employed to synthesize the first strand of cDNA. Quantitative real-time PCR was employed to amplify the full-length coding sequence of the target genes using specific primers.: *GAPDH-*F: 5′- AGG TCG GTG TGA ACG GAT TTG -3′, *GAPDH-*R: 5′- TGT AGA CCA TGT AGT TGA GGT CA -3′; *HAS2-F*:5′-GCT GAG TCT GGG CTA TGC AA-3′, *HAS2-*R: 5′-ACC CCT GTA GAA GAG CTG GAT-3′; *FNFAIP6*-F: 5′-GCA GGC GTG TAC CAC AGA GAA G-3′, *FNFAIP6*-R: 5′-CTG GCT GCC TCT AAC TGC TTG TAG-3′.

The real-time PCR reaction system was composed of 10 µL of 10xFaste Universal SYBR Green Master (ROX), 2 µL of cDNA template, 0.8 µL of both the forward and reverse primers, and 6.4 µL of ddH20. The PCR was then conducted with the ABI Step One Plus.

The quantitative real-time PCR reaction system was composed of 10 µL of the 10xFaste Universal SYBR Green Master (Roche, Basel, Switzerland, Cat# 4913914001), the cDNA template was then added to the mixture of 2 µL. Then, 0.8 µL of both the forward primer and reverse primer were added. Finally, 6.4 µLddH20 was added. The PCR was then conducted with the ABI Step One Plus. Three replicates of the 2^−ΔΔCt^ method were employed to determine the relative expression of each gene.

### 2.10. Statistical Analysis

For the purpose of the experimental statistics of oocyte maturation rate, the number of COCs per culture should not be lower than 30. Fluorescence intensity statistical experiments require a minimum of 15 oocytes per group for accurate results. At least three independent experiments were conducted for each analysis. The results of the study were expressed as means ± SEM for each group. Data were analyzed by t-test, provided by GraphPad Prism9.00 software (GraphPad, CA, USA). Statistical significance was indicated by asterisks, with one asterisk (*) denoting *p* < 0.05, two asterisks (**) denoting *p* < 0.01, three asterisks (***) denoting *p* < 0.001, and four asterisks (****) denoting *p* < 0.0001.

## 3. Results

### 3.1. Inhibition of Arp2/3 Complex Affects Goat Oocyte Maturation

**Figure 1:**
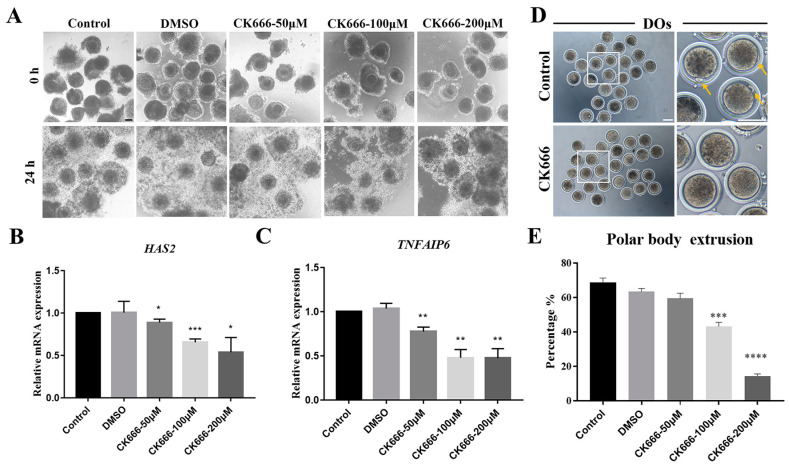
Inhibition of Arp2/3 complex affects goat oocyte maturation. (**A**) A selection of images depicting the expansion of cumulus clouds. Bar = 100 μm. (**B**,**C**) The expression of genes associated with cumulus expansion was observed. *, *p* < 0.05; **, *p* < 0.01; ***, *p* < 0.001. (**D**) DIC images of oocytes from the control group and those exposed to CK666 for 24h show different results. Yellow arrow indicated PBI. Bar = 100 μm. (**E**) The rate of polar body extrusion was compared between the control group and the CK666-exposed group after 24h of culture. ***, *p* < 0.001; ****, *p* < 0.0001.

The purpose of this study was to explore the potential role of Arp2/3 complex during goat oocyte maturation by using the Arp2/3 complex inhibitor CK666. We firstly investigated the oocyte maturation following different concentrations (50 μM, 100 μM, and 200 μM) of CK666 treatment. To conduct this study, the control group was established as an IVM medium without any other reagents, while the DMSO group was an IVM medium with 2‰ DMSO. This treatment occurred after 24 h of in vitro culture, and most oocytes should have reached the metaphase II (MII) stage by that point. Cumulus cells expansion was one of the hallmarks of oocyte maturation; thus, we first observed the expansion ability of cumulus cells. CK666 exposure resulted in numerous poor-quality cumuli, as opposed to the control and DMSO groups which had mostly good-quality cumuli. The expression levels of genes linked to cumulus expansion were examined in order to verify this. As shown in Figure 1B and Figure 1C, after exposure to CK666, the expression of genes related to cumulus expansion significantly decreased in comparison to the control (*HAS*, control: 1.00 vs. DMSO: 0.97 ± 0.13, *p* > 0.05; control: 1.00 vs. CK666-50 μM: 0.89 ± 0.04, *p* < 0.05; control: 1.00 vs. CK666-100μM: 0.65 ± 0.04, *p* < 0.001; control: 1.00 vs. CK666-50 μM: 0.54 ± 0.17, *p* < 0.05. *TNFAIP6*, control: 1.00 vs. DMSO: 1.04 ± 0.06, *p* > 0.05; control: 1.00 vs. CK666-50 μM: 0.78 ± 0.05, *p* < 0.01; control: 1.00 vs. CK666-100 μM: 0.48 ± 0.09, *p* < 0.01; control: 1.00 vs. CK666-200 μM: 0.47 ± 0.11, *p* < 0.01). The initial polar body extrusion was utilized to evaluate the influence of CK666 exposure on oocyte maturation. Research revealed that CK666 exposure decreases the first polar body extrusion. The statistical data showed that, when compared with the control group (68.67 ± 4.51%, n = 192 oocytes), the rate of polar body extrusion significantly decreased in 100 μM and 200 μM CK666 exposed oocytes (CK666-100μM: 43.10 ± 4.23%, *p* < 0.001, n = 132 oocytes; CK666-200 μM: 14.45 ± 1.65%, *p* < 0.001, n = 97 oocytes;). The data collected showed no significant difference in the two groups of individuals who were treated with DMSO or 50 μM CK666 (DMSO: 63.44 ± 3.13%, *p* > 0.05, n = 147 oocytes; CK666-50 μM: 59.52 ± 5.11%, *p* > 0.05, n = 126 oocytes) (Figure 1E). Overall, the results of the experiment pointed to the necessity of the Arp2/3 complex for the maturation of goat oocytes. When the complex was inhibited, the maturation process was significantly impeded.

### 3.2. Localization of Arp2 in Goat Oocytes

**Figure 2:**
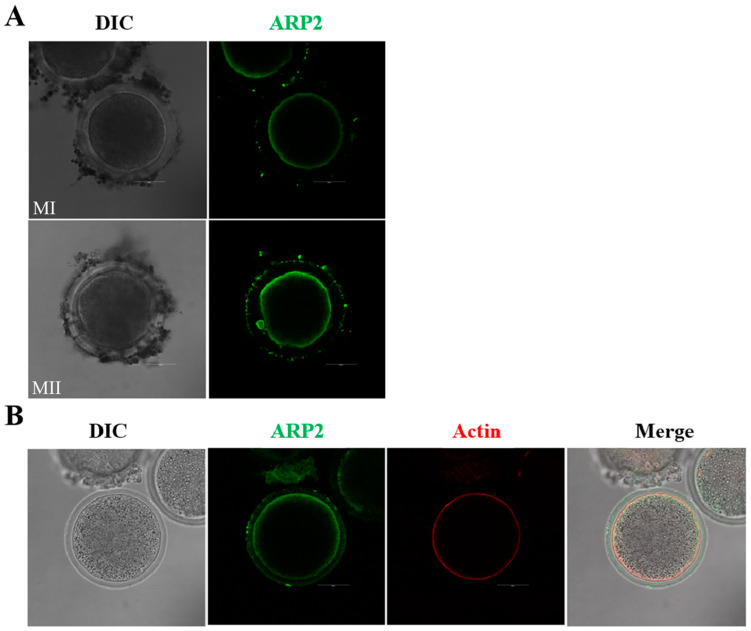
Localization of Arp2 in goat oocytes. (**A**) The subcellular localization of Arp2 in goat oocytes at MI and MII stages. Arp2 was found in greater abundance at the cortex. Green, Arp2-antibody. Bar = 50 μm. (**B**) Co-staining of oocytes for Arp2 and F-actin. Arp2 was present in the cortical region and was overlapping with F-actin. Green, Arp2-antibody; Red, F-actin. Bar = 50 μm.

By examining the subcellular localization of Arp2, we were able to gain a better understanding of the Arp2/3 complex. Arp2 is a subunit of this complex, and is expressed in goat oocytes. As shown in Figure 2A, Arp2 accumulates at the oocyte cortex during the metaphase I (MI)and MII stages. We also co-stained Arp2 with phalloidin in order to better visualize the actin cytoskeleton, as illustrated in Figure 2B, the study demonstrated that the signals of Arp2 and F-actin overlap at the cortex of the goat oocytes. The localization of Arp2 suggested that the Arp2/3 complex may be involved in the actin dynamics of goat oocyte meiosis.

### 3.3. Inhibition of Arp2/3 Complex Affects Actin Assembly in Goat Oocytes

**Figure 3:**
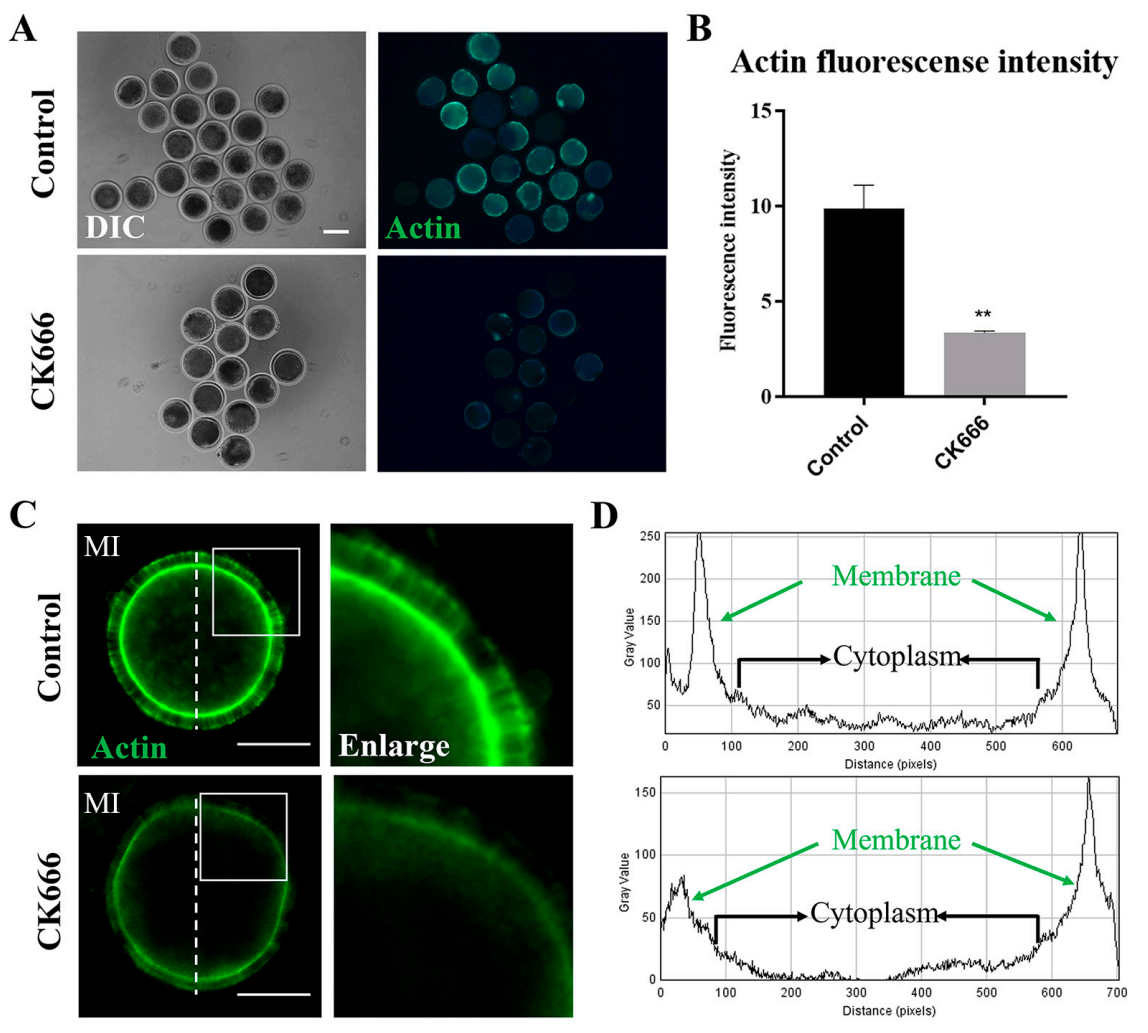
Inhibition of Arp2/3 complex affects actin assembly in goat oocytes. (**A**) A selection of images of F-actin in control and CK666-exposed oocytes. Green, F-actin. Bar = 100 μm. (**B**) The fluorescence intensity of F-actin signals was quantified in oocytes that were either control or CK666-exposed. **, *p* < 0.01. (**C**,**D**) The data presented in the graphs demonstrated the fluorescence intensity of actin filaments in oocytes that were exposed to either a control or CK666. The brightness of the pixels was measured along the lines drawn across the oocytes.

The Arp2/3 complex is a key actin nucleator that is known to co-localize with actin. The role of the Arp2/3 complex in oocyte meiosis could be mediated by its effects on the actin cytoskeleton, as suggested by the different organization of F-actin observed after phalloidin-FITC staining. Taking into account the considerable influence of 100 μM CK666 on oocyte meiosis, we chose to use 100 μM CK666 in our subsequent studies. To ensure that the single variable in the COCs culture process was kept constant, 1‰ DMSO was added to the control group. As presented in Figure 3A, the assembly of F-actin was found to be impaired in oocytes exposed to CK666, as evidenced by the weak fluorescence signal. This is in contrast to control oocytes, where F-actin was found to be equally concentrated on the plasma membranes (9.88 ± 2.74, n = 101 vs. 3.37 ± 0.15, n = 49, *p* < 0.01; Figure 3B). Confirmation of our research was shown through fluorescence plot profiling (Figure 3C,D), indicating the Arp2/3 complex’s role in the actin dynamics that facilitate goat oocyte maturation.

### 3.4. Inhibition of Arp2/3 Complex Affects Spindle Morphology and Chromosome Alignment in Goat Oocytes

**Figure 4:**
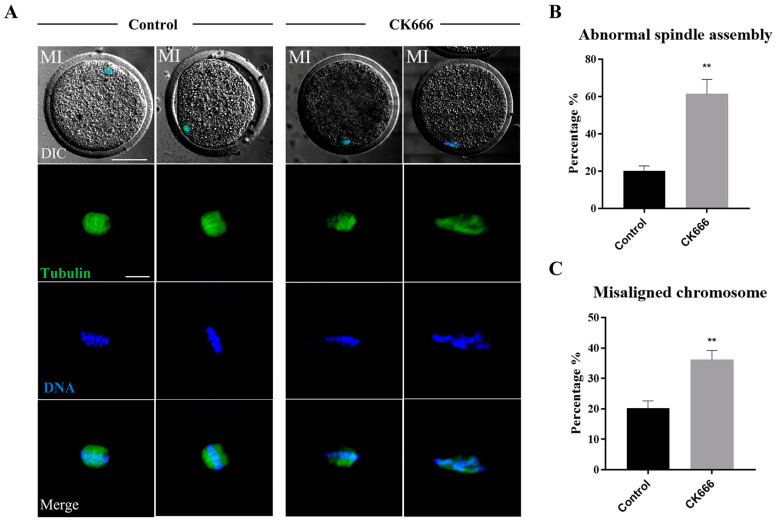
Inhibition of Arp2/3 complex affects spindle morphology and chromosome alignment in goat oocytes. (**A**) A selection of images of spindle morphology in the control and CK666-exposed oocytes. The spindles morphology of the oocytes in the CK666 exposure group exhibited a variety of defects when compared to the control group, with the chromosomes being severely misaligned. Green: tubulin; Blue, DNA. Bar = 50 μm and 20 μm. (**B**) An appreciable divergence was identified in the fraction of abnormal spindle morphology between the control group and the CK666-exposed group. **, *p* < 0.01. (**C**) An appreciable divergence was identified in the percentage of chromosome misalignment between the control group and the CK666-exposed group. **, *p* < 0.01.

We studied the spindle morphology and chromosomal arrangement of goat oocytes at the MI stage, with a special consideration of the role of F-actin in chromosome positioning and movement. As seen in Figure 4A, the control group oocytes demonstrated the anticipated cylindrical spindles, with chromosomes arranged in a typical manner at the equatorial plate. After the CK666 treatment, the regular cylindrical spindles became difficult to identify. Many oocytes displayed defects such as multipolarity, non-aggregation, and irregular shapes. Moreover, the degree of misalignment in chromosome arrangement was also significantly increased due to the abnormality of spindle shape. Data from the statistical analysis revealed that the blockade of the Arp2/3 complex caused an increase in the number of oocytes with defective spindles and misaligned chromosomes (the rate of aberrant spindles: control group vs. CK666 group: 20.25 ± 5.12%, n = 87 vs. 61.50 ± 15.50%, n = 61, *p* < 0.01) (Figure 4B); (the rate of misaligned chromosomes: control group vs. CK666 group: 20.25 ± 4.79%, n = 87 vs. 36.25 ± 5.91%, n = 61, *p* < 0.01) (Figure 4C). Research demonstrated that the Arp2/3 complex had an effect on goat oocyte maturation, as indicated by modifications in spindle morphology and chromosome positioning.

### 3.5. Inhibition of Arp2/3 Complex Affects Mitochondrial Functions in Goat Oocytes

**Figure 5:**
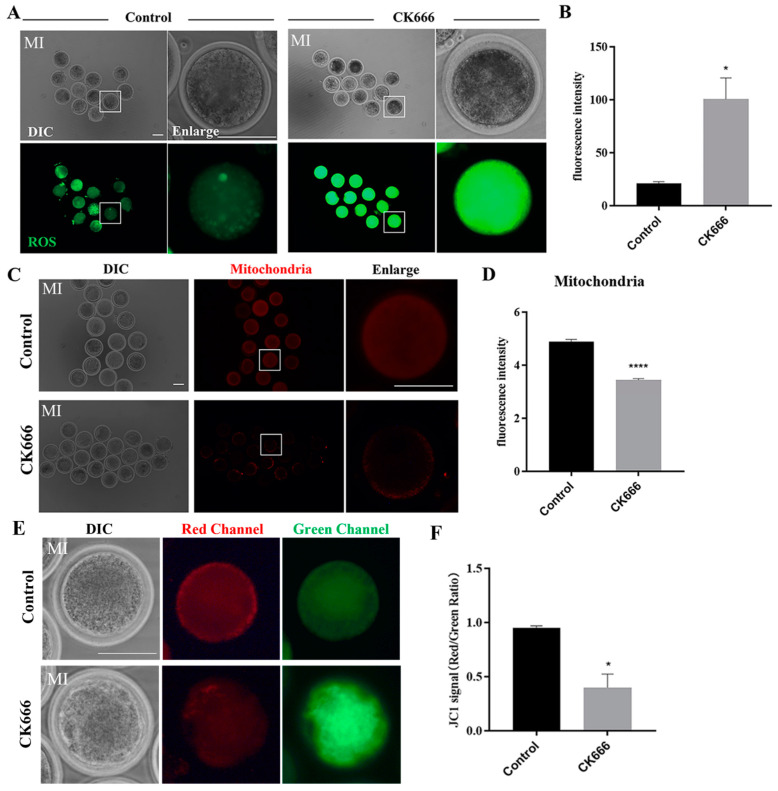
Inhibition of Arp2/3 complex affects mitochondrial functions in goat oocytes. (**A**) A selection of images of ROS fluorescence intensity in the control and CK666-exposed oocytes. Green: ROS. Bar = 100 μm. (**B**) Analysis of fluorescent intensities of ROS revealed a distinction between the control group and CK666-exposed group. *, *p* < 0.05. (**C**) Representative images of mitochondria in the control and CK666-exposed oocytes. Bar = 100 μm. (**D**) Analysis of the relative fluorescence intensity of mitochondria revealed a distinction between the control group and CK666-exposed group. ****, *p* < 0.0001. (**E**) The typical image for JC1 green channel and red channel in the control group and CK666 exposed group. Bar = 100 µm. (**F**) CK666 exposure results in a significant alteration of the JC1 signal (red/green ratio) when compared to the control group. *, *p* < 0.05.

Mitochondria play a central role in oxidative metabolism and are the primary source of reactive oxygen species (ROS). If mitochondrial function is impaired, ROS levels will rise. Actin are necessary for proper mitochondrial function. We sought to determine the potential role of Arp2/3 complex in goat oocyte maturation by investigating the levels of ROS in MI stage oocytes of control and CK666 groups. As Figure 5A, our findings demonstrated that CK666 treatment resulted in increased ROS signal compared with the control group, as supported by statistical analysis (control group vs. CK666 group: 21.36 ± 2.44, n = 45 vs. 100.81 ± 34.50, n = 36, *p* < 0.05) (Figure 5B). We next examined mitochondrial distribution using Mito-Tracker staining. As shown in Figure 5C, the results of the study showed that CK666 exposure caused mitochondria to mainly accumulate in the cortical region of oocytes (control group vs. CK666 group: 4.88 ± 0.20, n = 94 vs. 3.45 ± 0.09, n = 75, *p* < 0.0001) (Figure 5D). Finally, by examining the mitochondrial membrane potential (MMP), we found that the exposure of CK666 caused changes in MMP, as shown by JC-1 staining. As shown in Figure 5E, the fluorescence intensity of the JC-1 red channel was lower in the experimental group in comparison to the control group. This was also supported by the red/green fluorescence intensity ratio. (Control group vs. CK666 group: 0.95 ± 0.03, n = 40 vs. 0.40 ± 0.21, n = 36, *p* < 0.05) (Figure 5F). The study’s results imply that the Arp2/3 complex has an impact on mitochondrial function during goat oocyte meiosis.

### 3.6. Inhibition of Arp2/3 Complex Produces Apoptosis in Goat Oocytes

**Figure 6:**
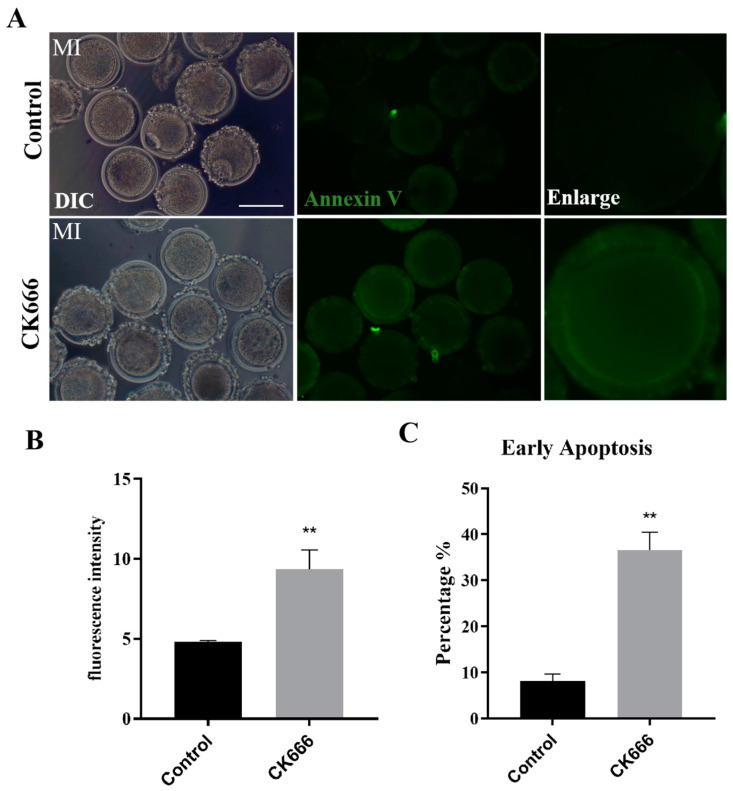
Inhibition of Arp2/3 complex produces apoptosis in goat oocytes. (**A**) A selection of images of Annexin-V fluorescence intensity in the control group and CK666 exposed group. Green: Annexin-V. Bar = 100 μm. (**B**) Analysis of fluorescent intensity of Annexin-V revealed a distinction between the control group and CK666- exposed group. **, *p* < 0.01. (**C**) The proportion of oocytes undergoing apoptosis was calculated in the control group and CK666- exposed group. **, *p* < 0.01.

The decrease in MMP is an irreversible event in the early stage of apoptosis, and excessive ROS could induce apoptosis. We next used Annexin-V staining to examine the apoptotic status of CK666-exposed oocytes. We classified the stained oocytes into two groups: those with a visible green staining on the cell membrane, signifying early apoptosis, and those without any specific staining on the cell membrane, indicating that they had not undergone early apoptosis. As per Figure 6A, the results showed that Annexin-V signals were hardly detected in the control group. However, they were evidently present on the membrane of CK666-exposed oocytes. This was supported by the statistical analysis (control group vs. CK666 group: 4.80 ± 0.19, n = 77 vs. 9.36±2.08, n = 44, *p* < 0.01) (Figure 6B). We conducted a count of the number of early apoptosis oocytes in the control group and the treatment group, a significantly higher proportion of early apoptosis was observed in CK666-exposed oocytes compared with controls (8.16 ± 2.58%, n = 77 vs. 36.59 ± 6.77%, n = 44, *p* < 0.01) (Figure 6C). The Arp2/3 complex seems to be involved in early apoptosis in goat oocytes.

## 4. Discussion

This study aimed to investigate the role of the Arp2/3 complex in goat oocyte meiosis. The findings suggest that the Arp2/3 complex is necessary for the maintenance of cytoskeletal meiotic dynamics, such as F-actin assembly and spindle organization in goat oocytes. Additionally, these cytoskeletal dynamics are essential for the regulation of mitochondrial distribution and function, including mitochondrial activity and redox homeostasis, which are critical for the meiotic maturation of goat oocytes.

All eukaryotic cells contain the Arp2/3 complex, which is necessary for the generation of branched actin networks. This complex is essential for the successful operation of many cellular processes [19]. CK666 is a small molecule inhibitor of the Arp2/3 complex which binds to Arp2 and Arp3, thus preventing the rearrangement of conformation that is necessary for activating the Arp2/3 complex [20,21]. The Arp2/3 complex is known to play a role in cell motility. However, its precise function in goat oocyte maturation is not well understood. In this study, we used the chemical inhibitor CK666 to investigate the role of the Arp2/3 complex in goat oocyte maturation. The effects of Arp2/3 complex on cumulus cell expansion and polar body extrusion were first detected. These two processes are considered important indicators of goat oocyte maturation. The CK666 treatment was found to have a noteworthy impact in lowering the prevalence of polar body extrusion and cumulus cell expansion that were inadequate. This is likely due to the reduced expression of genes involved in cumulus cell expansion (HAS2 and TNFAIP6) [22,23]. This research mirrors the contribution of Arp2/3 complex to meiosis in mouse and pig oocytes [14,18]. Nevertheless, our results contrast to some extent with those reported in mouse oocytes. Unlike the findings from mouse oocytes, CK666-treated goat oocytes can be divided asymmetrically as expected, with only a slight presence of large polar bodies. The divergence in the results is believed to be caused by the dissimilar positioning of the germinal vesicle in goat oocytes and mice. In comparison to mouse oocytes, the germinal vesicle of goat oocytes is generally eccentric, as a consequence, the radial migration of the MI spindle is shortened. This result is in agreement with the research conducted on Arp2/3 in porcine oocytes. Studies show that the Arp2/3 complex is an indispensable factor in the correct progression of meiotic maturation in goat oocytes.

The Arp2 subunit is an essential element of the Arp2/3 complex and is present in high amounts at the cortex of goat oocytes, where it interacts with F-actin. This arrangement of Arp2 is comparable to that of somatic cells or oocytes from other species, as has been documented in prior research [14,18,24,25]. It is hypothesized that the Arp2/3 complex could be implicated in actin-related activities during the meiotic process of goat oocytes, as evidenced by the presence of Arp2 in the area. 

To confirm our hypothesis, we then tried to explore how the Arp2/3 complex is involved in actin assembly in oocytes. Our findings demonstrated that the suppression of the Arp2/3 complex caused the breakdown of F-actin in both the cortex and the interior of goat oocytes. F-actin play a significant role in the meiosis of female mammalian oocytes, for example, it can regulate the cortical polarization of oocytes, polar body excretion, chromosome segregation and capture during oocyte meiosis [1,3]. The N-WASP protein, located upstream of the Arp2/3 complex, is essential for the successful polar body extrusion and spindle migration that takes place during porcine meiotic maturation [26]. The WHAMM protein, which is responsible for the activation of the Arp2/3 complex, is essential for the migration of peripheral spindles and the unequal division of cytokinesis during the maturation of mouse oocytes [27]. The control of Arp2/3 complex in goat oocytes is thought to be regulated through an actin-dependent pathway. Actin cage, a type of spindle actin, is necessary for the proper relocation of spindles and the segregation of chromosomes. This protein is similar in shape to a spindle and is necessary for these processes to occur [28]. Consistently, recent studies have shown that WHAMM is required for spindle actin formation and chromosome alignment. Our data also showed that inhibiting the Arp2/3 complex disrupts spindle organization and chromosome arrangement, which can lead to aneuploidy and abnormal cell division.

Mitochondria play a role in ATP production in cells by providing the necessary environment for this process to take place. F-actin plays a vital role in ensuring the proper distribution and function of mitochondria during oocyte meiotic maturation [29]. In oocytes, the meiotic spindle is a structure composed of microtubules that attach to homologous chromosomes during meiosis, this process requires a large amount of ATP [30]. We wondered if the Arp2/3 complex would affect mitochondrial function in oocytes, since it changes microfilament viability. We also wondered if abnormal oocyte spindle morphology after CK666 treatment is related to mitochondrial damage. Furthermore, when mitochondrial function is impaired, ROS may accumulate beyond the ability of cells to scavenge them, causing oxidative stress [31]. Thus, the results of the study showed that the inhibition of the Arp2/3 complex affected the functions of mitochondria in goat oocytes. This was evident through an increase in ROS levels, disrupted distribution of mitochondria, and decreased mitochondrial membrane potential. Simultaneously, mitochondria are important for various oocyte functions, such as energy production, cell signaling, and cell death [32]. If there is an imbalance in mitochondrial fission and fusion processes, this can cause mitochondrial dysfunction, the production of ROS, and apoptosis [33]. Simazine exposure has been shown to cause mitochondrial dysfunction and oxidative stress, both of which are associated with early oocyte apoptosis [34]. PFOA-induced oocyte deterioration was caused by mitochondrial dysfunction and apoptosis in the F1 offspring [35]. A wealth of research has demonstrated that mitochondrial damage to oocytes can trigger apoptosis, adversely affect meiotic progression, and reduce oocyte quality. Lastly, our findings illustrated that apoptosis was dramatically altered after Arp2/3 complex inhibition. Altogether, the Arp2/3 complex was found to be associated with mitochondria dysfunction and apoptosis in goat oocytes.

## 5. Conclusions

Collectively, we provide a body of evidence demonstrating that the Arp2/3 complex was a critical regulator for goat oocyte maturation through its regulation of cytoskeleton dynamics and mitochondrial function.

## Data Availability

All data generated or analyzed during this study are included in this published article.
